# Supplementary figures and images for: Longitudinal IgG antibody responses to Plasmodium vivax blood-stage antigens during and after acute vivax malaria in individuals living in the Brazilian Amazon
Source: PLoS Negl Trop Dis. 2022 Nov 23;16(11):e0010773. doi: 10.1371/journal.pntd.0010773 (PMC9728838; doi:10.1371/journal.pntd.0010773)

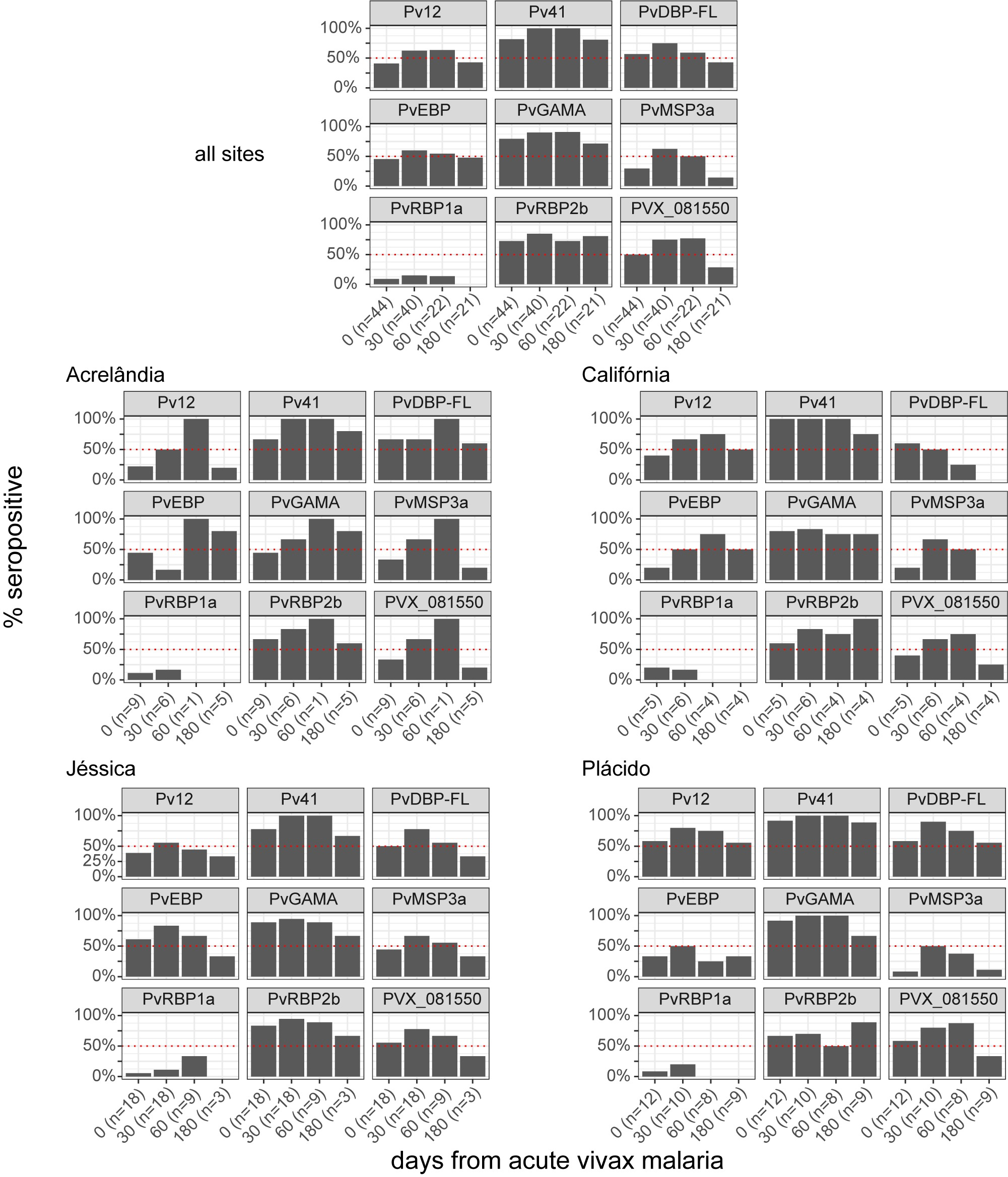

Supplement: S1 Fig — Percent seropositive at each time point by study site for each antigen. Number (n) in parenthesis indicates number of subjects with available plasma samples at each time point. Dotted red line indicates 50% seroprevalence. (TIF) [file pntd.0010773.s001.tif]
