# Supplementary material for: Longitudinal IgG antibody responses to Plasmodium vivax blood-stage antigens during and after acute vivax malaria in individuals living in the Brazilian Amazon
Source: PLoS Negl Trop Dis. 2022 Nov 23;16(11):e0010773. doi: 10.1371/journal.pntd.0010773 (PMC9728838; doi:10.1371/journal.pntd.0010773)
Supplement: S1 Table — Details for the recombinant proteins used in the multiplex immunoassays used in the study. (DOCX) [file pntd.0010773.s002.docx]

**Table S1.** Recombinant proteins used in the study.

| **PlasmoDB accession #** | **Protein** | **Description** | **Plasmid source (addgene)** | **MW** | **His tag**  **(4.2 kDa)** | **rat CD4 tag**  **(20.4 kDa)** | **estimated total MW (kDa)** | **References** |
| --- | --- | --- | --- | --- | --- | --- | --- | --- |
| PVX_097720 | MSP3a (full length) | Merozoite Surface Protein 3a | #68509 | 90.6 | Yes | Yes | ~95 | [1] |
| PVX_088910 | PvGAMA  (full length) | *Plasmodium vivax*  GPI-anchored micronemal antigen | #68522 | 80.8 | Yes | No | ~85 | [1, 2] |
| PVX_113775 | Pv12  (full length) | *Plasmodium vivax* 12 | #68516 | 38.6 | Yes | No | ~43 | [1, 2] |
| PVX_110810 | DBP  (full length) | Duffy binding protein ectodomain | #68528 | 117.1 | Yes | Yes | ~142 | [1] |
| PVX_000995 | Pv41  (full length) | *Plasmodium vivax* 41 | #68519 | 44.1 | Yes | Yes | ~68 | [1] |
| PVP01_0102300 | EBP (DBP 2) | Erythrocyte Binding Protein/Duffy Binding Protein 2 | Gene synthesized in Twist Biosciences | 95.1 | Yes | No | ~120 | this study and  [1] |
| PVX_081550 | StAR-related lipid transfer protein, putative  (full length) | StAR-related lipid transfer protein putative | #68532 | 56.9 | Yes | Yes | ~81 | [1] |
| PVX_094255 | PvRBP2b | *Plasmodium vivax* reticulocyte binding protein 2b | - | 152.8 | No | No | ~153 | [3, 4] |
| PVX_098585 | PvRBP1a | *Plasmodium vivax* reticulocyte binding protein 1a | - | 117.9 | No | No | ~118 | [4] |
| - | Cd4 | rat Cd4 domain 3 and 4 tag | - | 20.3 | No | No | ~24.5 | Rayner Lab |

**References**

1. Crosnier C, Wanaguru M, McDade B, Osier FH, Marsh K, Rayner JC, et al. A library of functional recombinant cell-surface and secreted P. falciparum merozoite proteins. Mol Cell Proteomics. 2013;12(12):3976-86. Epub 2013/09/18. doi: 10.1074/mcp.O113.028357. PubMed PMID: 24043421; PubMed Central PMCID: PMCPMC3861738.

2. Crosnier C, Staudt N, Wright GJ. A rapid and scalable method for selecting recombinant mouse monoclonal antibodies. BMC Biol. 2010;8:76. Epub 2010/06/08. doi: 10.1186/1741-7007-8-76. PubMed PMID: 20525357; PubMed Central PMCID: PMCPMC2898661.

3. Gruszczyk J, Lim NT, Arnott A, He WQ, Nguitragool W, Roobsoong W, et al. Structurally conserved erythrocyte-binding domain in Plasmodium provides a versatile scaffold for alternate receptor engagement. Proc Natl Acad Sci U S A. 2016;113(2):E191-200. Epub 2015/12/31. doi: 10.1073/pnas.1516512113. PubMed PMID: 26715754; PubMed Central PMCID: PMCPMC4720341.

4. Gruszczyk J, Kanjee U, Chan LJ, Menant S, Malleret B, Lim NTY, et al. Transferrin receptor 1 is a reticulocyte-specific receptor for Plasmodium vivax. Science. 2018;359(6371):48-55. Epub 2018/01/06. doi: 10.1126/science.aan1078. PubMed PMID: 29302006; PubMed Central PMCID: PMCPMC5788258.
